# A multi-parameter tunable plasmon modulator

**DOI:** 10.1038/s41598-023-38799-y

**Published:** 2023-07-17

**Authors:** Xuefang Hu, Changgui Lu, Xiangyue Zhao, Yinwei Gu, Mengjia Lu, Dechao Sun

**Affiliations:** 1 College of Digital Technology and Engineering, Ningbo University of Finance & Economics, Ningbo, 315175 Zhejiang China; 2 Key Laboratory of Optical Information Detection and Display Technology of Zhejiang, Zhejiang Normal University, Jinhua, 321004 Zhejiang China; 3 Advanced Photonics Center, School of Electronic Science and Engineering, Southeast University, Nanjing, 210096 Jiangsu China

**Keywords:** Optical properties and devices, Nanophotonics and plasmonics

## Abstract

Multi-parameter control of light is a key functionality to modulate optical signals in photonic integrated circuits for various applications. However, the traditional optical modulators can only control one or two properties of light at the same time. Herein, we propose a hybrid structure which can modulate the amplitude, wavelength and phase of surface plasmon polaritons (SPPs) simultaneously to overcome these limitations. The numerical results show that when the Fermi level of graphene changes from 0.3 to 0.9 eV, the variation of optical transmission, wavelength and phase are 32.7 dB, 428 nm and 306°, respectively. The demonstrated structure triggers an approach for the realization of ultracompact modulation and has potential applications in the fields of optical switches, communications and photo-detection.

## Introduction

As the essential property of electromagnetic field, polarization, wavelength, amplitude and phase reflect a wealth of applications in optical perception and operation^1–4^. Effectively manipulating of these parameters at micro/nanoscales is of great significance and has been widely exploited in optical communication^5–8^, optical sensing^9–11^, photo-detection^7, 12^ and so on. Surface plasmon polaritons (SPPs), originating from the interaction between free electrons of metal and the electromagnetic waves, hold a more potential applications at micro/nanoscales because they can break the conventional diffraction limit^13, 14^. Herein, the active control of SPPs has attracted great attention of researchers^15, 16^.

Currently, much work has been done in multi-parameter modulation to meet the increasing demand for various application scenarios^17–19^. For example, Zhuang Ren’s group demonstrated an active and smart electro-optic THz modulator, which is based on a strongly correlated electron oxide vanadium dioxide (VO_2_). With milliampere current excitation on the VO_2_ thin film, the transmission, reflection, absorption and phase of THz waves can be modulated efficiently. In particular, the antireflection condition can be actively achieved and the modulation depth reaches 99.9%, accompanied by a 180° phase switching^20^. Ali Forouzmand’s group proposed an electrically tunable amplitude and phase modulators, which are designed by the hybridization of indium tin oxide (ITO) into a guided-mode resonance mirror. A gate-tunable amplitude modulator with a modulation depth as high as ∼ 0.80 is realized, and the phase variation of ∼ 210° is accomplished when the applied bias voltage alters from − 15 V to + 24 V^21^. However, these proposed modulators can only control one or two parameter of light despite its superior performance, which greatly hinders their widely application^22, 23^. Herein, a modulator which can control more properties of light will attract much attention of experts.

In this paper, a hybrid silicon-dielectric-graphene-grating structure is proposed, which can modulate the amplitude, wavelength and phase of SPPs simultaneously. The SPPs is stimulated by the grating and propagating on the graphene, the Fermi level of the graphene is controlled by the voltage applied between silicon substrate and graphene. The numerical results show that when the Fermi level changes from 0.3 to 0.9 eV, the variation of optical transmission, wavelength and phase are 32.7 dB, 428 nm and 306°, respectively. This structure has the potential application in the fields of optical switches, communications and photo-detection^24, 25^.

## Model and analysis

**Figure 1:**
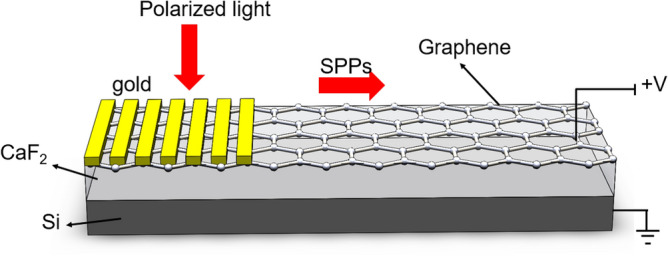
3D Schematic diagram of silicon-dielectric-graphene-grating structure. The polarized light incident on the gold grating and excites the SPPs propagating along the graphene surface. The Fermi level of the graphene is controlled by the voltage applied between silicon substrate and the graphene.

In Fig. 1, we illustrate the three-dimensional (3D) schematic diagram of our designed hybrid silicon-dielectric-graphene-grating structure. A TM polarized light incidents on the gold grating and excites the SPPs propagating along the graphene surface. The Fermi level of the graphene is controlled by the voltage applied between silicon substrate and the graphene, leading to the amplitude, wavelength and phase of SPPs modulated simultaneously. Because the calcium fluoride (CaF_2_) exhibit an excellent performance such as low leakage current, high dielectric strength and low amounts of defects, we select the CaF_2_ as the dielectric layer, which could improve the performance of modulator effectively^26^.

To understand the relationship between the applied voltage and the carrier density in our model, a theoretical relationship between them is shown in Eq. (1), where e, ɛ_0_ and ɛ_r_ are the charge constant, the permittivity of vacuum and the relative permittivity of CaF_2_ respectively, *V* and *d* are the voltage and thickness of CaF_2_. The relation between Fermi level and the carrier density is presented in Eq. (2), where $\hslash$, V_f_ and n_g_ are the reduced Planck constant, Fermi velocity and carrier density respectively, where V_f ≈_10^6^ m/s^27–29^. Herein, the Fermi level of the graphene can be modulated by the voltage effectively and dynamically^30, 31^.

1 $${n}_{g}=\frac{{\varepsilon }_{0}{\varepsilon }_{r}V}{ed},$$

2 $${E}_{f}=\hslash {V}_{f}\sqrt{\pi {n}_{g}}.$$

**Figure 2:**
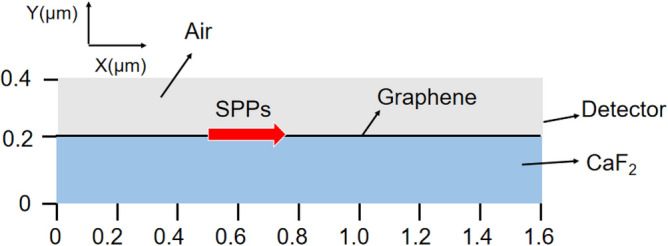
2D simulation model of proposed hybrid silicon-dielectric-graphene-grating structure, the port mode is used to excite the SPPs.

The proposed structure is simulated with the finite element method (FEM), and the two-dimensional (2D) simulation model is shown in Fig. 2. The port mode is used to excite the SPPs, (The grating is just used to excite the SPPs, so it is unnecessary to take the grating into consideration in simulation since the excitation efficiency is insignificant here and other excitation method can be applied.) and then the characteristics of the SPPs are analyzed by changing the Fermi level of graphene (The details about the simulation are shown in section of method). Firstly, the electric field distribution and optical transmittance are used to reflect the modulation about the amplitude. Secondly, the change in wavelength can be observed in the distribution of electric field too. Finally, the phase distribution of this model is presented. Thus, the demonstrated simulation model can vividly reflect the modulation about the amplitude, wavelength and phase.

For the model of anisotropic graphene, its out-of-plane permittivity is set to 2.5, and the in-plane conductivity can be obtained from random-phase approximation, including the effect of finite temperature (T = 300 K):

3 $$\begin{aligned} \sigma_{g} & = \frac{{2ie^{2} K_{B} T}}{{\pi \hbar^{2} (\omega + i\tau^{ - 1} )}}In\left[ {2\cosh \left( {\frac{{E_{F} }}{{2K_{B} T}}} \right)} \right] \\ & \quad + \frac{{e^{2} }}{4\pi \hbar }\left\{ { - \frac{i}{2}In\frac{{(\hbar \omega + 2E_{F} )^{2} }}{{(\hbar \omega - 2E_{F} ) + (2K_{B} T)^{2} }} + \frac{\pi }{2} + \arctan \left( {\frac{{\hbar \omega - 2E_{F} }}{{2K_{B} T}}} \right)} \right\}. \\ \end{aligned}$$

Here σ_g_ and k_B_ are the conductivity of graphene and the Boltzmann’s constant. E_f_ and ɷ are the Fermi energy level and radian frequency, respectively. The carrier relaxation time τ = μE_f_/ev_f_^2^, where the Fermi velocity v_f_ = 10^6^ m/s and carrier mobility μ = 10,000 cm^2^/(V s). The effective permittivity of graphene ɛ_g_ can be described by means of the following expression:

4 $$\varepsilon_{g} = 1 + \frac{{i\sigma_{g} }}{{\omega \varepsilon_{0} t_{g} }},$$

where ɛ_0_ and t_g_ = 0.33 nm represent the vacuum permittivity and thickness of graphene respectively^27^.

## Results and discussion

### Amplitude

**Figure 3:**
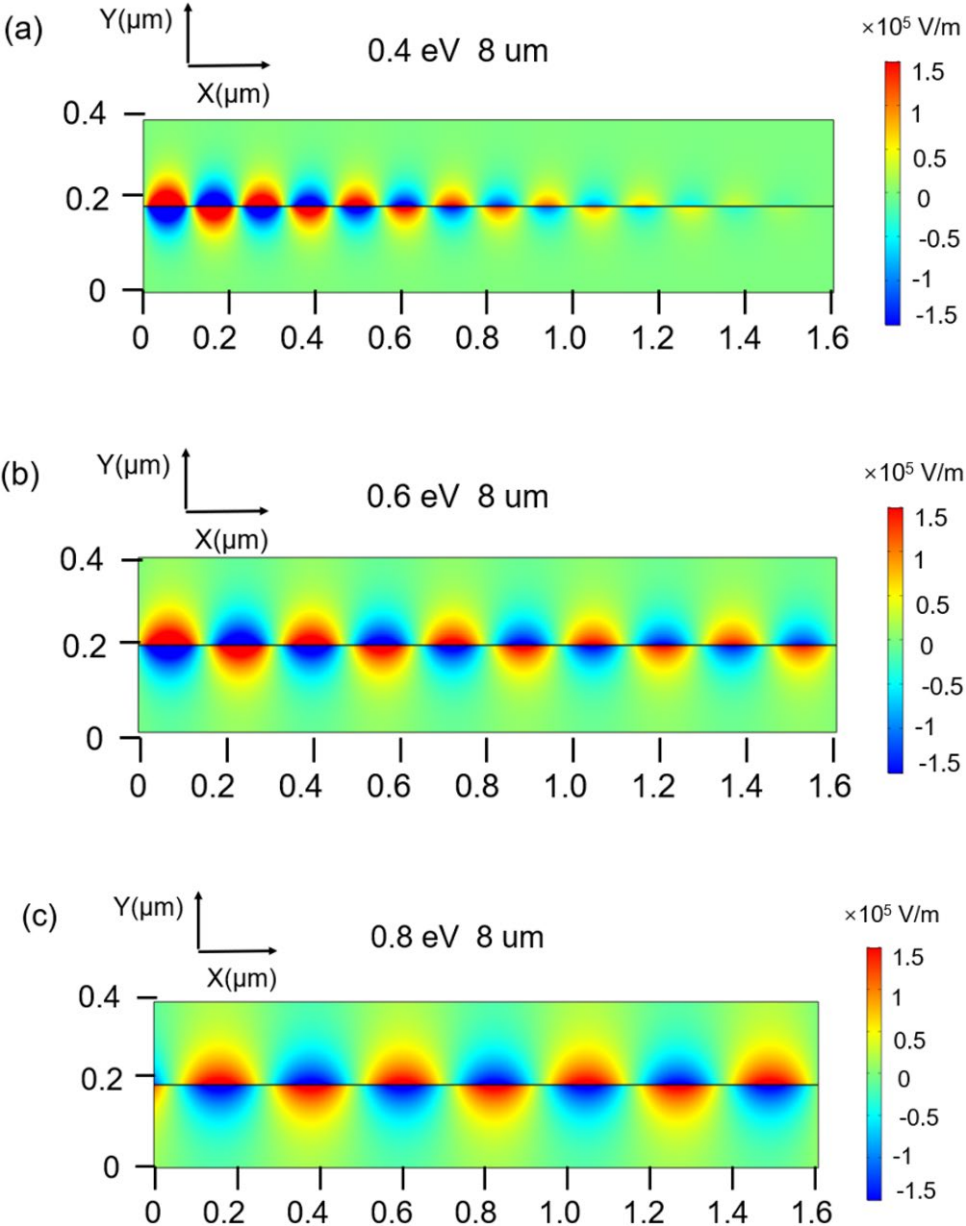
The y component of electric field distribution when the Fermi level of graphene is (**a**) 0.4 eV (**b**) 0.6 eV (**c**) 0.8 eV.

Figure 3 depicts the y component of electric field at different Fermi levels, and the working wavelength is fixed at 8 μm. Figure 3a is the y component of the electric field when the Fermi level is 0.4 eV. The distribution of electric field is the typical SPPs and just presents a normal attenuation. The y component of the electric field is drawn in Fig. 3b when the Fermi level is 0.6 eV as the voltage increases. It can be obtained that the strength of the electric field is enhanced and the loss of SPPs is decreased, resulting in a longer SPPs propagating distance. In addition, the wavelength of propagating SPPs is also increased. The y component of the electric field when the Fermi level is 0.8 eV is shown in Fig. 3c, and the change in amplitude and the wavelength is more obvious. The distribution of electric field at different Fermi level proves that the proposed silicon-dielectric-graphene-grating structure can control the amplitude and the wavelength of SPPs effectively via changing the applied voltage.

**Figure 4:**
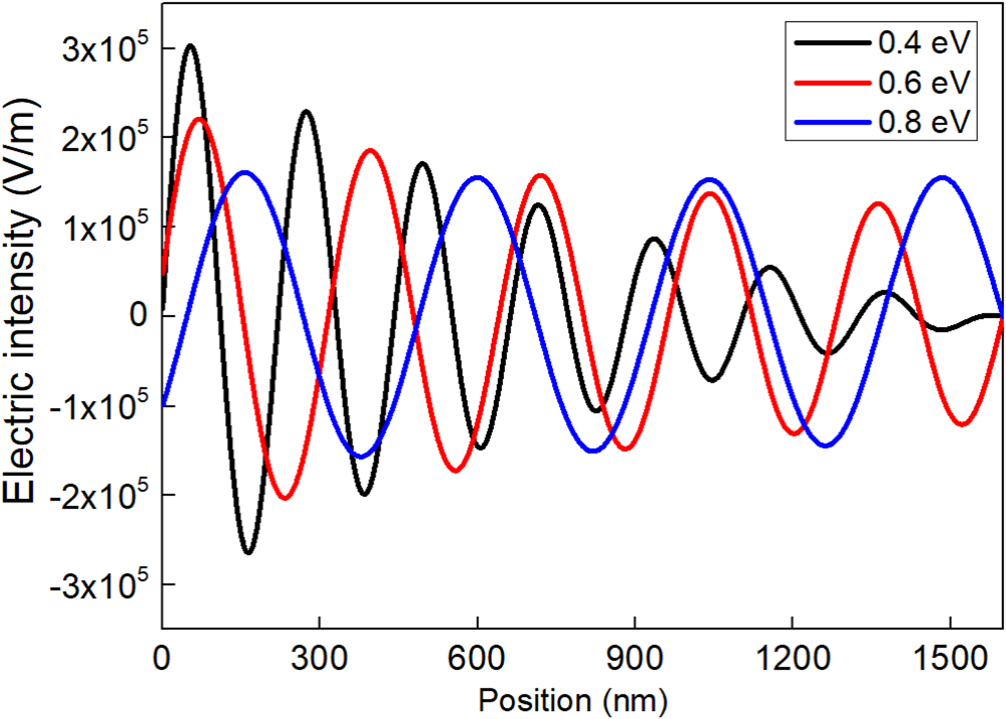
The relationship between electric intensity and Fermi levels of graphene.

Figure 4 shows the relationship between electric intensity and the Fermi levels of graphene. It can be seen that the electric intensity of fourth peak at the Fermi level of 0.4 eV is 1.24 × 10^5^ V/m, while it is 1.37 × 10^5^ V/m when the Fermi level increases to 0.6 eV. The electric field intensity reaches 1.53 × 10^5^ V/m as the Fermi level increases to 0.8 eV, and it has changed about 23.3%. It can be concluded that with the increase of the Fermi level, the amplitude of the SPPs increases gradually.

**Figure 5:**
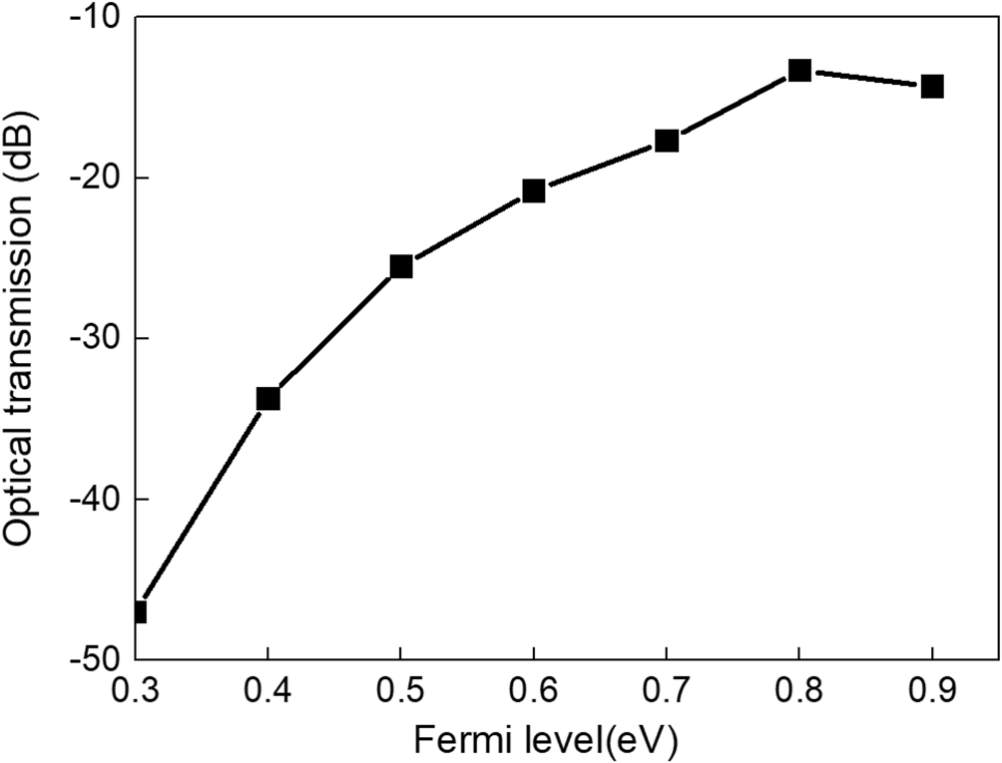
The relationship between optical transmission and the Fermi levels of graphene.

Figure 5 shows the relationship between optical transmittance and different Fermi levels of graphene. It can be obtained that the optical transmission increases obviously as the Fermi level increases, because the propagation losses is decreased owing to the increase of carrier density. The optical transmission changes about 32.7 dB (− 47 dB at 0.3 eV while − 13.3 dB at 0.8 eV). It further illustrates that our proposed structure can effectively control the amplitude of the propagating SPPs dynamically by changing the Fermi level of graphene.

### The wavelength

**Figure 6:**
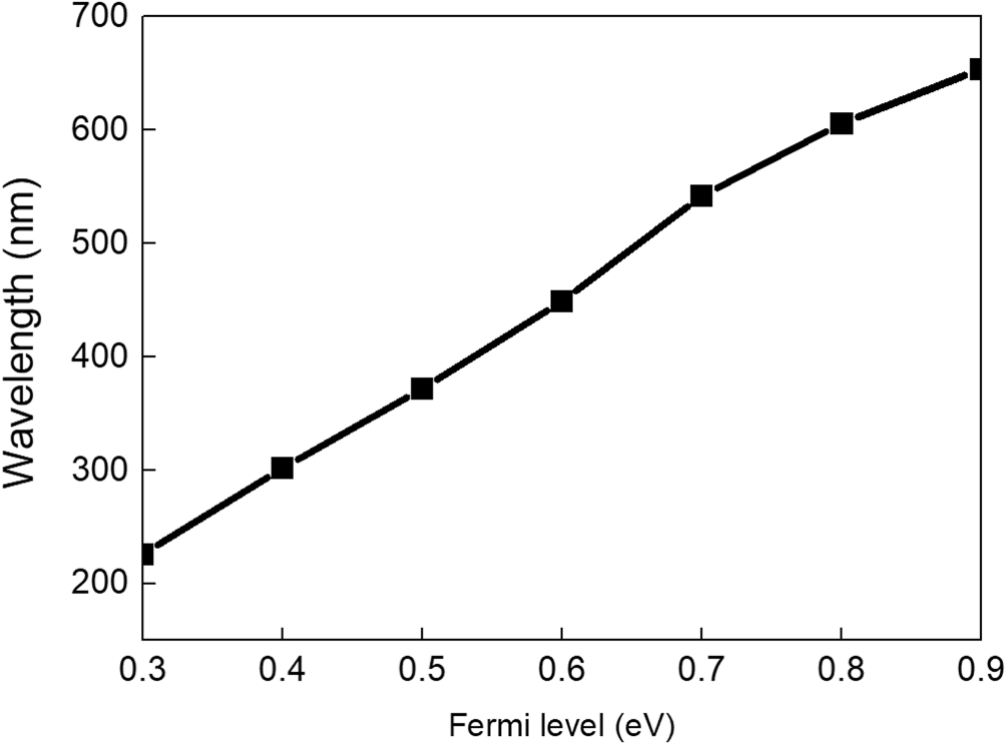
The relationship between wavelengths of SPPs and Fermi levels.

Figure 6 is the relationship between wavelength of SPPs and the Fermi levels of graphene. It can be obtained from picture that the wavelength of the propagating SPPs increases when the Fermi level increases, owing to the wavelength of graphene plasmon that is in direct proportional to the Fermi level of the graphene^32^. The wavelength changes by 428 nm when the Fermi level of graphene increase from 0.3 to 0.9 eV (226 nm at 0.3 eV while 654 nm at 0.9 eV). The results verify that the wavelength of the SPPs can also be modulated by the demonstrated structure effectively.

### The phase

**Figure 7:**
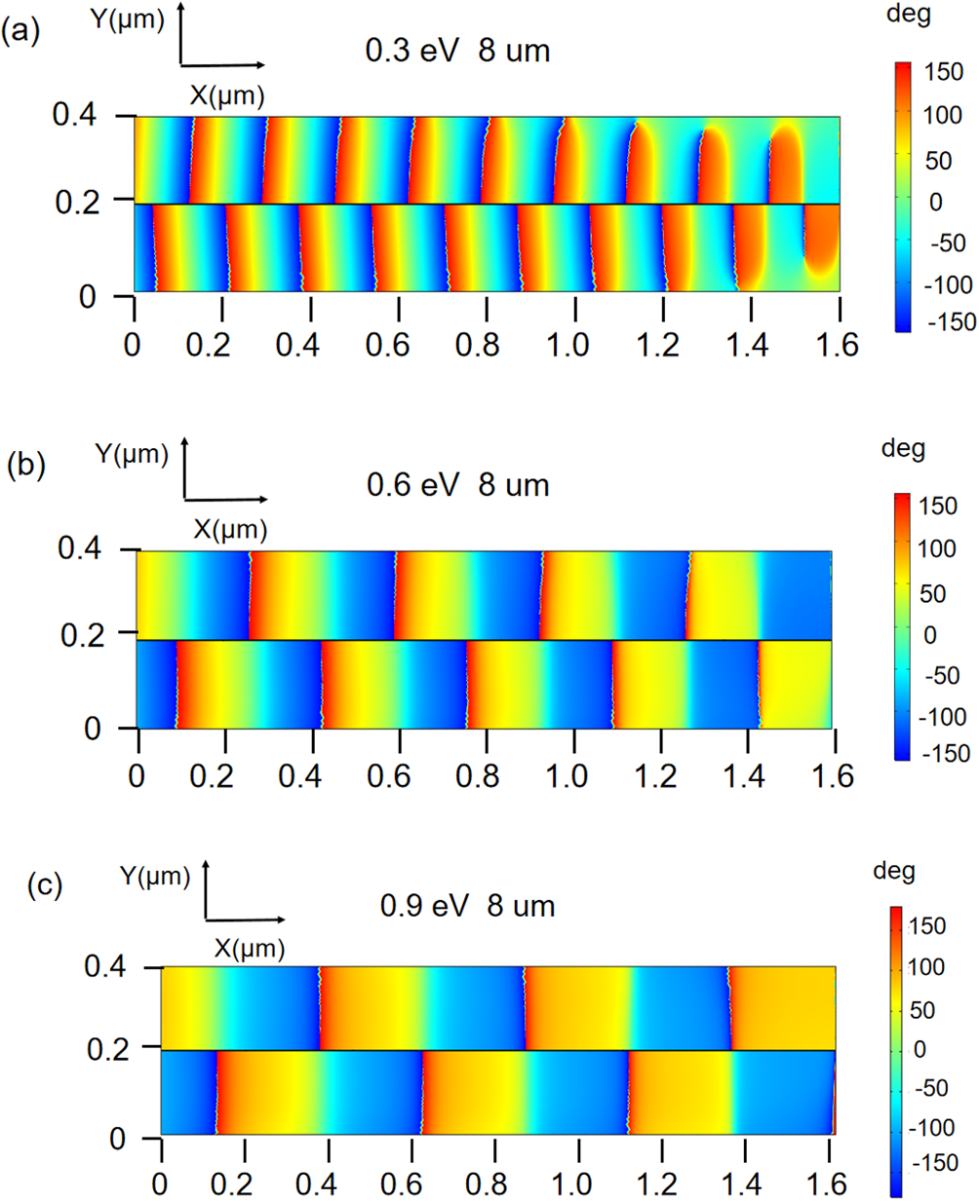
The phase distribution at different Fermi levels (**a**) 0.4 eV (**b**) 0.6 eV (**c**) 0.9 eV.

Figure 7 shows the x component of the phase distribution when the graphene is of different Fermi levels. It can be clearly seen that the phase present various distribution for different Fermi levels, which verifies that our proposed structure can also be utilized to control the phase of SPPs dynamically.

**Figure 8:**
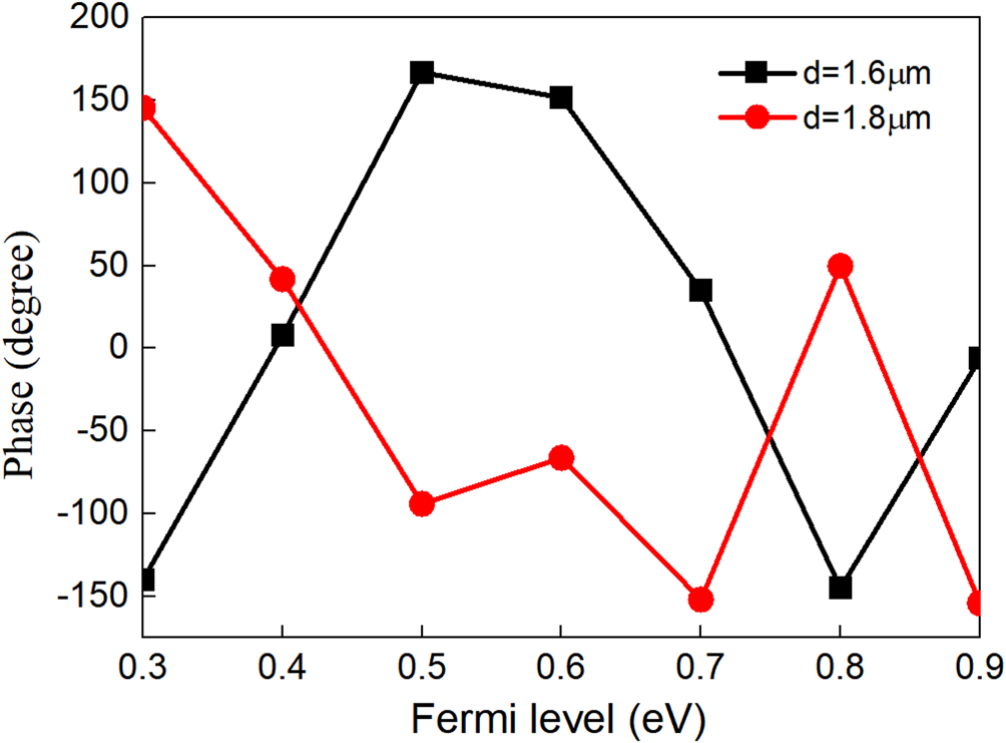
The relationship between phase at right port and the Fermi levels of graphene when the propagation lengths are 1.6 μm and 1.8 μm, respectively.

The phase of SPPs can be affected by both the refractive index and the propagation length of graphene due to the change of optical path. Figure 8 is the relationship between the phase at the right port and the Fermi levels of graphene when the propagation length *d* is 1.6 μm and 1.8 μm, respectively (the operating wavelength is fixed at 8 μm). The phase is − 139° when the Fermi level of graphene is 0.3 eV, and the phase is 167° when the Fermi level increases to 0.5 eV at the propagation length of 1.6 μm. The phase changes at right port changes can reach to 306°. (While the phase is 145° when the Fermi level of graphene is 0.3 eV, and the phase is − 154° when the Fermi level increases to 0.9 eV at the propagation length of 1.8 μm. The phase changes 299°). Thus, the amplitude, wavelength and phase of propagating SPPs in our model can be simultaneously controlled through the Fermi level of graphene by changing the applied voltage, which will have a potential application in modern integrated optics and communication^33^.

## Conclusion

In conclusion, we have demonstrated a silicon-dielectric-graphene-grating hybrid structure. The amplitude, wavelength and phase of SPPs can be modulated simultaneously when the SPPs is propagating along the graphene. The numerical results show that when the Fermi level of graphene changes from 0.3 to 0.9 eV, the optical transmission, wavelength and phase are changed by 32.7 dB, 428 nm and 306°, respectively. The realization of multi-parameter modulation in SPPs will have a promising application in the field of optical communication, sensor and photo-detection.

## Methods

The simulation has been performed using the commercial finite element method (FEM), trial version of software “COMSOL Multiphysics 5.5”. In simulation, the scattering boundary condition and user-defined port are used. The module of radio frequency is used to investigate the relationship between voltage and optical transmission, wavelength and phase. The surface conductivity model and the transitional boundary condition are used in the model of graphene. In addition, the permittivity of CaF_2_ is set as 6.76. The mesh size is 1/5 of one wavelength.

## Data Availability

The data relative to the experiments discussed in this work are available upon reasonable request from the corresponding author Xuefang Hu.
